# Exploration of binding and inhibition mechanism of a small molecule inhibitor of influenza virus H1N1 hemagglutinin by molecular dynamics simulation

**DOI:** 10.1038/s41598-017-03719-4

**Published:** 2017-06-19

**Authors:** Shanshan Guan, Tianao Wang, Ziyu Kuai, Mengdan Qian, Xiaopian Tian, Xiuqi Zhang, Yongjiao Yu, Song Wang, Hao Zhang, Hao Li, Wei Kong, Yaming Shan

**Affiliations:** 1 National Engineering Laboratory for AIDS Vaccine, School of Life Sciences, Jilin University, Changchun, Jilin China; 2 Key Laboratory for Molecular Enzymology and Engineering, The Ministry of Education, School of Life Sciences, Jilin University, Changchun, Jilin China; 3 Laboratory of Theoretical and Computational Chemistry, Institute of Theoretical Chemistry, Jilin University, Changchun, Jilin China; 4 College of Biology and Food Engineering, Jilin Engineering Normal University, Changchun, Jilin China

## Abstract

Influenza viruses are a major public health threat worldwide. The influenza hemagglutinin (HA) plays an essential role in the virus life cycle. Due to the high conservation of the HA stem region, it has become an especially attractive target for inhibitors for therapeutics. In this study, molecular simulation was applied to study the mechanism of a small molecule inhibitor (MBX2329) of influenza HA. Behaviors of the small molecule under neutral and acidic conditions were investigated, and an interesting dynamic binding mechanism was found. The results suggested that the binding of the inhibitor with HA under neutral conditions facilitates only its intake, while it interacts with HA under acidic conditions using a different mechanism at a new binding site. After a series of experiments, we believe that binding of the inhibitor can prevent the release of HA1 from HA2, further maintaining the rigidity of the HA2 loop and stabilizing the distance between the long helix and short helices. The investigated residues in the new binding site show high conservation, implying that the new binding pocket has the potential to be an effective drug target. The results of this study will provide a theoretical basis for the mechanism of new influenza virus inhibitors.

## Introduction

Influenza virus is the causative agent of influenza, an infectious disease which usually leads to symptoms such as high fever, cough, headache, muscle and joint pain, sore throat, nasal discharge, and even a fatal illness similar to pneumonia^1–4^. Influenza viruses are divided into three types, type A, type B and type C, with influenza A virus presenting serious threats to public health worldwide due to its high mutation rate^5–7^. At present, two classes of drugs are approved by the Food and Drug Administration for treatment or chemoprophylaxis of influenza: matrix protein 2 (M2) inhibitors amantadine and rimantadine and the neuraminidase (NA) inhibitors (NAIs) oseltamivir and zanamivir^8, 9^. However, with the wide use of these drugs, drug-resistant strains have appeared in succession^10^. Therefore, new antiviral targets with novel inhibition mechanism need to be developed.

Hemagglutinin (HA), a viral receptor-binding and membrane-fusion glycoprotein involved in the invasion of influenza into host cells, plays an essential role in the life cycle of influenza A virus^3, 11^. HA is a trimer of identical subunits, each of which consists of a variable membrane-distal receptor-binding globular domain (HA1) and a more conserved membrane-proximal helix-rich stem structure (HA2)^12^. Under acidic conditions, the residues on the outer surface of HA1 can be protonated easily, which leads to the large gathering of positive charges on the surface of HA^13, 14^. With gradually increased charges, disaggregation of HA first occurs as the positive charges repel each other, followed by the entry of water into the interior of the protein causing further structural changes of HA2^13^. In the HA2 subunit, one short and one long α-helical segment are connected by a loop (helix-loop-helix structure)^3, 14, 15^. The N-terminal segment next to the short helix is a fusion peptide consisting of 20 amino acids, while the C-terminal end of the long helix is anchored to the viral membrane^3^. Acidic pH conditions in the endosome can trigger conformational changes involving a folding of the loop connecting the two helices of HA2 into a new longer helix (coiled-coil structure) of the HA ectodomain (loop-to-helix transition) and further trigger its membrane fusion capacity^1, 3^. From the above observations, stabilizing the “helix-loop-helix” structure of HA2 may be considered crucial for preventing the loop-to-helix transition^11, 15^.

The sequence and structure of the HA stem loop region are well known to be highly conserved, which makes it an especially attractive target for entry inhibitors for therapeutics^16^. Currently, many anti-flu drug target studies have focused on this functional membrane protein. For instance, Arnab and colleagues successfully obtained two novel promising compounds, MBX2329 and MBX2546, using a high-throughput screening assay (HTS), a way to screen novel compounds from a chemical library for inhibitory activity of some functional protein, and demonstrated their potency in the inhibition of HA^16, 17^. Other HA-inhibiting compounds such as BMY 27709^18^, 180299 (podocarpic acid derivative)^19^ and tert-butyl hydroquinone (TBHQ)^20^ have also been confirmed. These compounds can serve as starting points for the development of a therapeutic agent. Therefore, studying the inhibition mechanism of novel compounds in theory is necessary.

It has been confirmed that MBX2329^16^, the compound mentioned above, could inhibit HA of A/Washington/10/2008(H1N1) and A/Florida/21/2008(H1N1) at similar levels (IC_50_ of 0.29 µM and 0.3 µM). However, it’s still not clear about the theoretical basis of its inhibition mechanism. In order to explore molecular inhibition mechanism of new HA inhibitors, in this study, molecular docking, molecular dynamics simulation and the Molecular Mechanics/Poisson-Boltzmann Surface Area (MM-PBSA) approach were applied to study the detailed mechanism of HA inhibition of this representative compound MBX2329, which hereafter is referred to as INT for convenience^16^. The findings of this study will be useful for future exploration of efficient drug targets and provide theoretical insight into a new mechanism of influenza virus inhibitors.

## Theory and Methods

### Preparation of initial complexes

HAs from experimental strains A/Washington/10/2008(H1N1) (HA_Washington_) and A/Florida/21/2008(H1N1) (HA_Florida_)^16^ were chosen for the preferred models to investigate the INT binding pose under neutral condition, and eventually, the results between experiment and simulation were compared. According to the high homology between HA_Washington_ and HA_Florida_, the structure of HA_Florida_ could be regarded as a repeated system in the following molecular simulations to ensure the accuracy of molecular dynamics sampling. The three-dimensional structures of the above HA were obtained via Swiss-modeling^21, 22^, a homology modeling procedure, since their structures have not been crystallized. The HA structure from the Research Collaboratory for Structural Bioinformatics Protein Data Bank (RCSB PDB ID: 4LXV^23^) was chosen as the template for homology modeling as its highly BLAST sequence alignment similarity (higher than 87%) to HA_Washington_’s or HA_Florida_’s (Fig. S1a). Procheck^24, 25^ was used to validate the rationality of homology models built with the Swiss model online. In order to perform a further investigation on inhibition mechanism under acidic condition, a third HA structure of strains 1918(H1N1) (RCSB PDB ID: 1RUZ^26^) was chosen for the model at a low-pH state according to the calculated pK_a_ results for its titratable residues as studied by Zhou *et al*.^27^. Protonation state of the titrable residues in 1RUZ is shown in Table S1. Energy minimizations were performed on all HA with GROMACS 4.5.2^28^ using steepest descent techniques before docking calculations. The structure of INT was optimized at the B3LYP 6–31 + G* level using the Gaussian 09 software^29^. Relevant structures are shown in Supplementary Materials Fig. S2.

AutoDock Vina^30^ was used for automatic placement of INT in the binding pockets of HAs to obtain the initial structure of INT-HA complexes. AutoDock Vina is a new program for molecular docking and virtual screening, which uses a sophisticated gradient optimization method in its local optimization procedure. For AutoDock Vina, a grid of 24 × 22 × 24 points in the x, y, and z-axis directions was built with a grid spacing of 1 Å, the center of which was treated as the mass center of one monomer of HA, and the exhaustiveness was set to 20^31^. The grid detail used in the docking simulations is shown in Fig. S3.

### Molecular dynamics simulation

The three molecular systems are listed in Table 1. All of the complex systems were subjected to molecular dynamics simulation in a periodic boundary condition using the GROMACS 4.5.2 software package^28^ with SPC (simple point charge) water model^32^. The Gromos 53 A6 force field^33^ was applied to describe both the receptors and ligand. The parameterization of the INT was produced by Automated Topology Builder (ATB) server^34^. To keep each system at an electrically neutral state, an appropriate amount of ions was added to randomly replace the water molecules (18 sodium ions for INT-HA_Washington_ complex, 15 sodium ions for INT-HA_Florida_ complex and 33 chloride ions for INT-HA_1ruz_ complex). First, energies of the complex systems were relaxed with steepest-descent energy minimization to ensure that the systems were without steric clashes or incorrect geometry. Thereafter, 500 ps NVT (constant Number of particles, Volume, and Temperature) and NPT (constant Number of particles, Pressure, and Temperature) were alternately run with position restraints on HA and INT to allow for relaxation of the solvent molecules in two phases. The solvent molecules were equilibrated with fixed protein at 300 K, taking the initial velocities from a Maxwellian distribution. Subsequently, the protein and inhibitor were relaxed step by step and heated up to 300 K. The long-range electrostatics were described with the particle mesh Ewald algorithm^35^ with interpolation order of 4, a grid spacing of 0.16 nm and the Coulomb cutoff distance of 1.0 nm. The LINCS (Linear Constraint Solver) algorithm was used to constrain all bonds. Temperature and pressure coupling types were set with V-rescale and Parrinello-Rahman, respectively^31, 36^. In the NVT ensemble, the temperature of the systems reached a plateau at the desired value (reference temperature = 300 K; time constant = 0.1 ps). In addition, the equilibration of pressure (reference pressure = 1.0 bar; time constant = 2.0 ps) was performed under the NPT ensemble. Finally, molecular dynamics simulations for collecting data with a time step of 2 fs and coordinates saved every 2 ps were initiated^29, 31^.

**Table 1:** Molecular systems.

Simulations	Time (ns)	Number of solvent	Number of ions	Simulation condition
INT-HA_Washington_	200	48393	18 NA^+^	Neutral condition
INT-HA_Florida_	100	44543	15 NA^+^	Neutral condition
INT-HA_1ruz_	200	62831	33 CL^−^	Acidic condition

### Calculation of binding free energy and ligand-residue interaction decomposition

In our study, the binding free energies were calculated using the MM-PBSA method^37–39^ supplied with the Amber 10 package^40^. A total number of 100 snapshots were chosen evenly from the molecular dynamics trajectory^31^. The MM-PBSA method can be conceptually summarized as:

1 $${{\rm{\Delta }}{\rm{G}}}_{{\rm{bind}}}={{\rm{\Delta }}{\rm{G}}}_{{\rm{complex}}}-[{{\rm{\Delta }}{\rm{G}}}_{{\rm{protein}}}+{{\rm{\Delta }}{\rm{G}}}_{{\rm{lig}}}]$$

2 $${{\rm{\Delta }}{\rm{G}}}_{{\rm{bind}}}={\rm{\Delta }}{\rm{H}}-T{\rm{\Delta }}S$$

where the *ΔH* of the system consists of the enthalpy changes in the gas phase upon complex formation (*ΔE*_*MM*_) and the solvated free energy contribution (*ΔG*_*sol*_), while *−TΔS* refers to the entropy contribution to the binding^31^. Equation (2) can then be approximated as shown in Eq. (3):

3 $${{\rm{\Delta }}{\rm{G}}}_{{\rm{bind}}}={{\rm{\Delta }}{\rm{E}}}_{{\rm{MM}}}+{{\rm{\Delta }}{\rm{G}}}_{{\rm{sol}}}-{\rm{T}}{\rm{\Delta }}{\rm{S}}$$

In addition, the change in flexibility of HA and inhibitor upon binding is similar in all complexes. Based on previous studies^41^, the entropy differences should be very small. Thus, the calculation of the solute entropy term was omitted in the present study^31, 42^.

*ΔE*_*MM*_ is the summation of the van der Waals (*ΔE*_*vdw*_) and the electrostatic (*ΔE*_*ele*_) interaction energies.

4 $${{\rm{\Delta }}{\rm{E}}}_{{\rm{MM}}}={{\rm{\Delta }}{\rm{E}}}_{{\rm{vdw}}}+{{\rm{\Delta }}{\rm{E}}}_{{\rm{ele}}}$$

In addition, *ΔG*_*sol*_, which denotes the solvation free energy, can be computed as the summation of an electrostatic component (*ΔG*_*ele,sol*_) and a nonpolar component (*ΔG*_*nonpolar,sol*_), as shown in Eq. (5). The nonpolar contribution to the free energy was calculated via γSASA, where SASA is the solvent-accessible surface area and γ is 0.0072 kcal/mol/Å^2^. The SASA was estimated to be a 1.4 Å solvent-probe radius. Referring to nonpolar solvation options the radii used in the MM-PBSA calculations are mbondi2.

5 $${\rm{\Delta }}{G}_{sol}=({\rm{\Delta }}{G}_{ele,sol})+({\rm{\Delta }}{G}_{nonpolar,sol})$$

The interactions between the inhibitor and each residue in the binding site were analyzed using the MM-PBSA decomposition process applied in the MM-PBSA module in Amber 10. The binding interaction of each ligand-residue pair includes three terms: the van der Waals contribution (*ΔE*_*vdw*_), the electrostatic contribution (*ΔE*_*ele*_) and the solvation contribution (*ΔE*_*sol*_). All energy components were calculated using the same snapshots as the free energy calculation^31, 42^.

## Results and Discussion

Influenza virions that are attached to the host cell membrane via HA are phagocytosed and gain entry into the cell through endocytosis^11^. During this endocytosis process, small molecules generally can enter the endosome at the same time^43^. Thus, the effective combination of Inhibitors of HA under neutral conditions outside the cell is crucial for the intake of INT. After entering into the endosome, HA will undergo large conformational changes under acidic conditions, which is aimed at promoting the membrane fusion between the virus and host cell^1, 3^. An efficient HA entry inhibitor must be able to stop the progression of changes in this molecule. In order to investigate if INT could stably bind with HA under neutral conditions and be efficiently carried into the endosome to exert its inhibition at low-pH conditions, the optimal binding mode, binding force and binding behavior of INT with HA were determined by a series of molecular dynamics simulations and MM-PBSA calculation.

### Analysis of reliability of investigated complex systems

**Figure 1:**
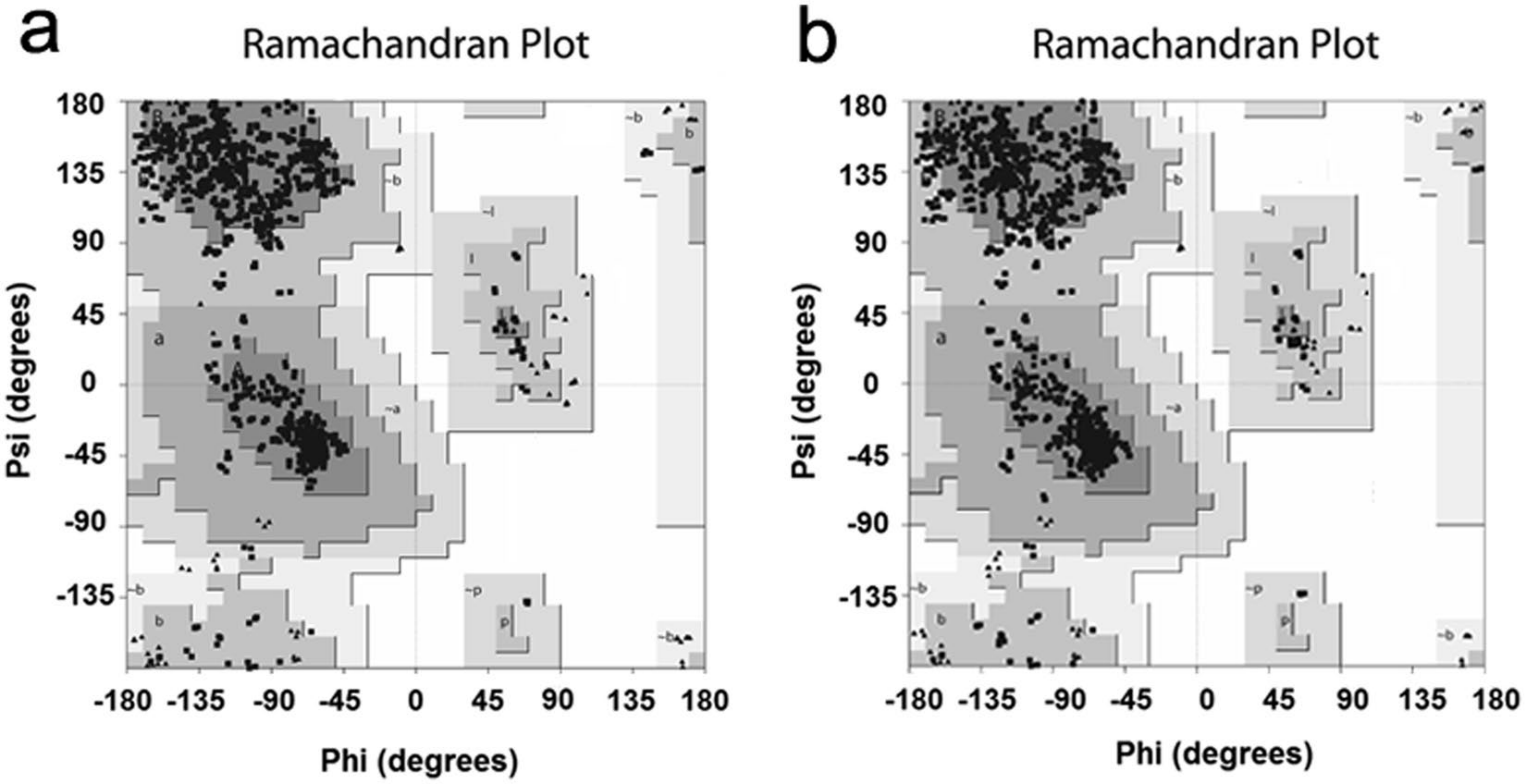
Ramachandran plot of modeled HA. (**a**) A/Washington/10/2008(H1N1) HA. (**b**) A/Florida/21/2008(H1N1) HA.

The validation was carried out using Ramachandran plot calculations computed with the Procheck^24, 25^ program by examining the detailed residue-by-residue stereochemical quality of HA structures, and the results are shown in Fig. 1. Altogether, 100% of the investigated residues were in allowed regions, which confirmed the availability of the modeled HA protein systems. AutoDock Vina was used for obtaining the initial structure of INT-HA complexes. The docking results were in agreement with those from the mutation experiments and molecular docking reported by Arnab *et al*.^17^, which indicated that the findings could be applied to the next molecular dynamics study. Relevant docking results (Fig. S4) and comparisons with previous results from Arnab *et al*. can be found in the Supplementary Materials.

**Figure 2:**
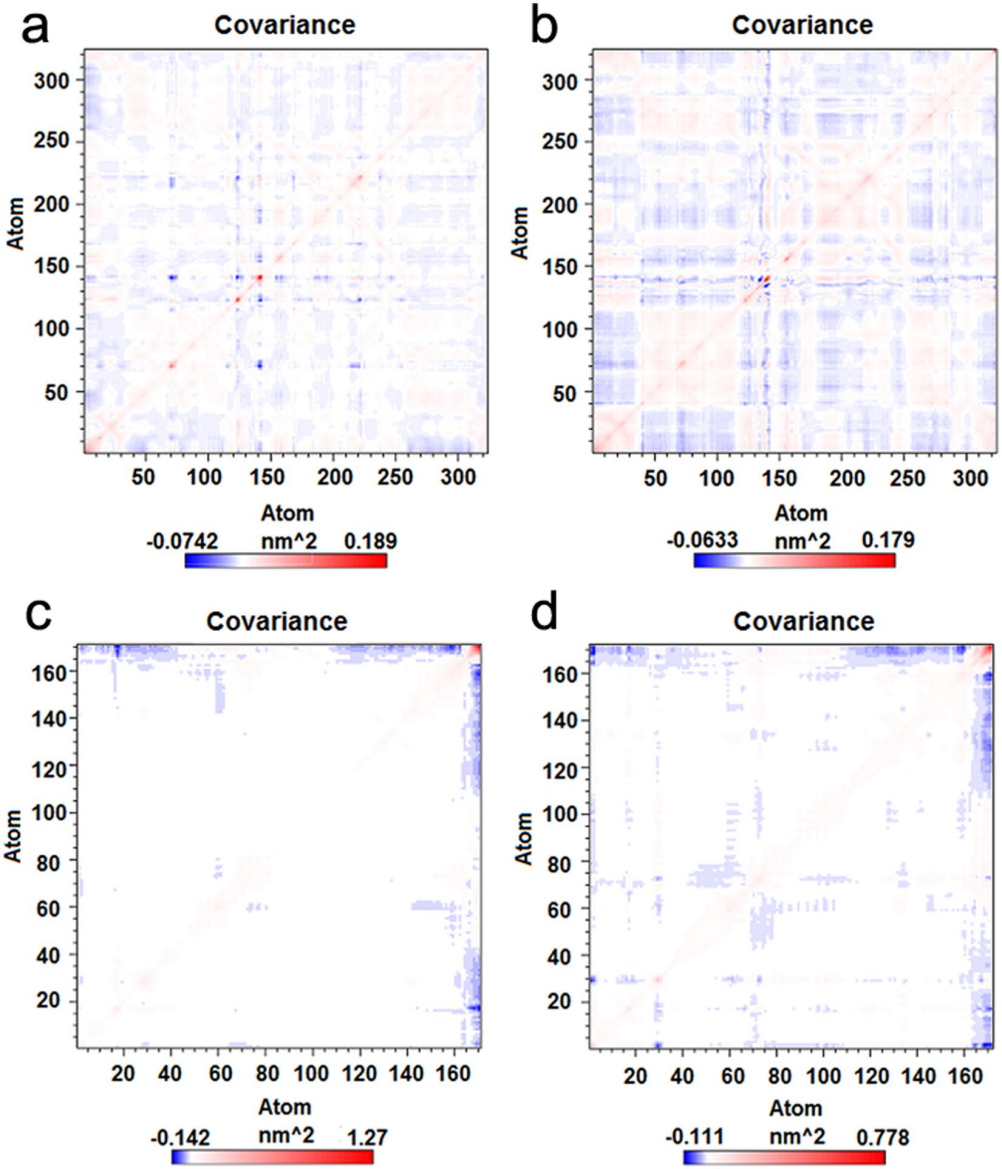
Cross-correlation matrix of the fluctuations of each of the x, y, and z coordinates of the Cα atoms of HA from their average during molecular dynamics simulations. (**a**) Cα atoms from HA1 of HA_Washington_. (**b**) Cα atoms from HA1 of HA_Florida_. (**c**) Cα atoms from HA2 of HA_Washington_. (**d**) Cα atoms from HA2 of HA_Florida_.

For the accuracy of dynamics sampling, the complex structure of INT-HA_Florida_ was regarded as a replicate system of INT-HA_Washington_ under the neutral condition molecular simulations. In order to monitor if two systems possess similar dynamics behaviors, the cross-correlations of their C_α_ atomic displacements, a parameter which can reflect the detailed atomic dynamic state, were analyzed during the simulations^29^. The relevant results are illustrated in Fig. 2. Highly positive regions (red) indicate a strong correlation in the movement of residues, whereas negative regions (blue) are associated with strong anti-correlated motion of the residues. The color depth of diagonal could represent the movement degree of atoms. As shown in Fig. 2a and b, the similarity could be observed in the behavior of C_α_ atoms from HA_Washington_ HA1 and HA_Florida_ HA1. Also the similar behavior could be discovered from C_α_ atoms from HA_Washington_ HA2 and HA_Florida_ HA2 as shown in Fig. 2c and d. As expected, no obvious differences in the movement of atoms occurred between the two complexes during the timescale of simulations. Moreover, according to the structures of both simulations, the conformations of two repeated simulations were found to be identical. Two independent dynamics samplings yielded similar results. Thus, in the following sections, results from only INT-HA_Washington_ are discussed.

### Binding mode of complex system under neutral conditions

**Figure 3:**
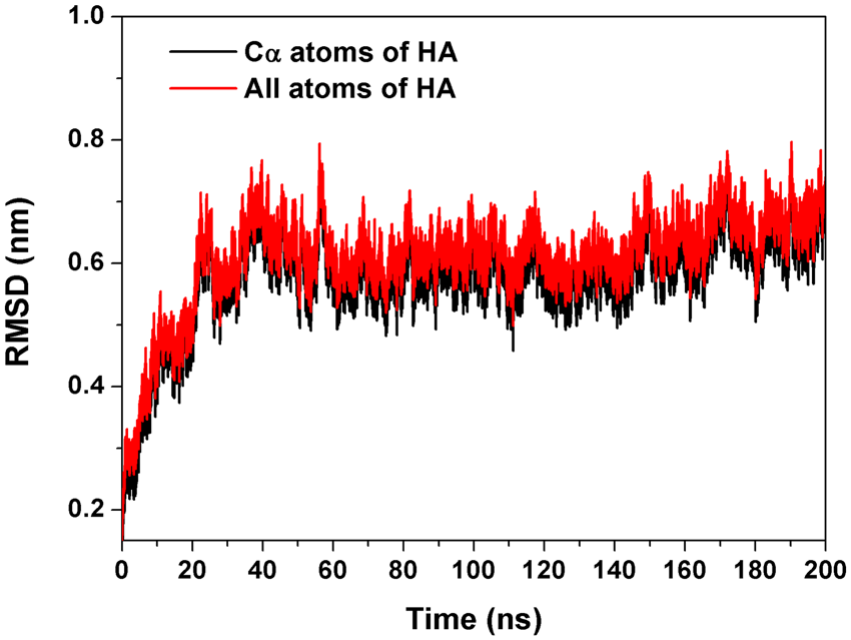
RMSD plot during molecular dynamics simulations.

For convenience, INT-HA_Washington_ is simplified as INT-HA in the following section. After 200 ns simulations, the root-mean-square deviation (RMSD) of backbone Cα atoms and all atoms of HA were first investigated to evaluate if the complex system could reach an equilibrium during the simulation^44^. As shown in Fig. 3, the RMSD curve of the INT-HA complex was found to reach the equilibrium at about 40 ns, suggesting that the structures could be applied to analyze the optimal binding mode between INT and HA^31^.

**Figure 4:**
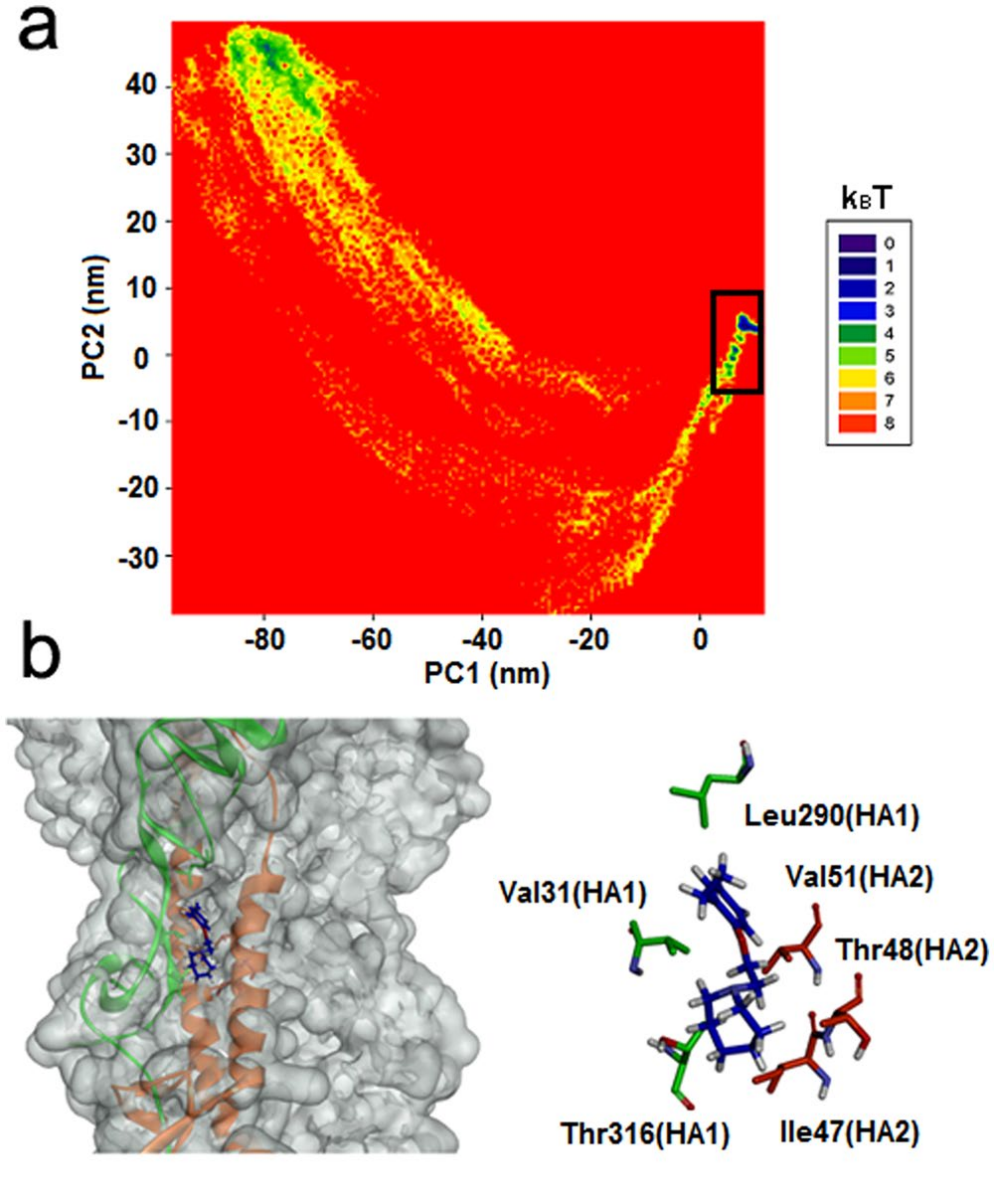
(**a**) Relative free energy surfaces along the first two principle components (PC-1, PC-2) of INT-HA under neutral conditions. (**b**) Predicted binding modes of INT-HA under neutral conditions.

In order to obtain the most stable complex structure, cluster analysis of INT-HA was investigated to determine the optimal binding mode. In addition, the comparison of free energy surfaces between the first 100 ns and further one with extended 100 ns were applied to confirm the convergence of complexes (Fig. S5). Cluster analysis is a method that can categorize similar low-energy conformations into the same designated area by analyzing the relative free energy surfaces along the first two principle components (PC-1, PC-2) of the complex during simulation^29, 33^. Two principal amounts (PC-1 and PC-2) were obtained as the reaction coordinate that renders the potential of the mean force (ΔF = −RT × lnP, where P is the relative probability in a region) free-energy plane^42^. The first few PCs describe the slow-motion modes which are related to the functional motions of a biomolecular system. In the cluster analysis plot (Fig. 4a), the conformations found in the blue area indicated more stable and lower energy states than those found in the red area. And these lower energy conformations generally could be chosen as the best analysis subjects for the binding modes. As shown in Fig. 4a, more than 80% of the conformations converged at the blue area, and the structure from this area could be applied to the binding mode analysis. The detailed binding mode of INT-HA based on the above analysis is shown in Fig. 4b. The results revealed that INT is bound in the pocket located on the interface between HA1 and HA2. This pocket is surrounded by the side chains of HA1-Val31, HA1-Leu290, HA1-Thr316, HA2-Ile47, HA2-Thr48 and HA2-Val51. The side chain of HA1-Val31 extends towards C9 of INT, and the side chain of HA1-Leu290 is close to C7 of INT. HA1-Thr316, HA2-Ile47, HA2-Thr48 and HA2-Val51 together approach the seven-member heterocycle of INT.

**Figure 5:**
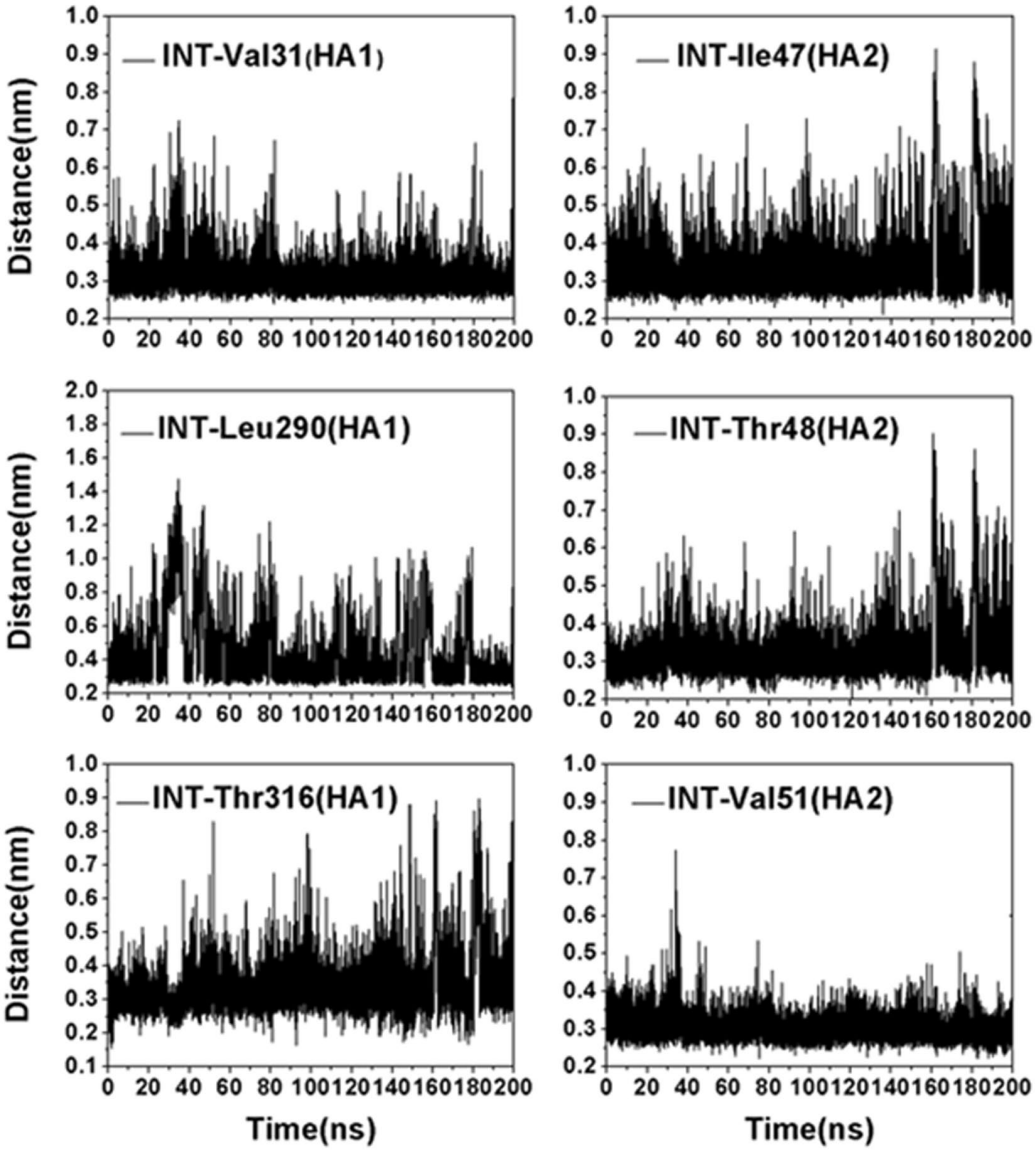
Distances between INT and residues (HA1-Val31, HA1-Leu290, HA1-Thr316, HA2-Ile47, HA2-Thr48 and HA2-Val51) around the binding site under neutral conditions.

In order to confirm if INT could stably bind with HA in this pocket during the simulation, the distances between the above residues and INT were calculated by using the distance analysis program of GROMACS. According to the corresponding results shown in Fig. 5, all of the distances monitored were around 0.3–0.4 nm during the 200 ns simulation, which indicated the accuracy of the binding pose. INT is a molecule with an aminoalkyl phenol ether scaffold^16^ and has no hydrogen donor or receptor. Thus, the stabilization of INT in the binding pocket most likely contributes to nonpolar interactions. It is well known that the van der Waals radius of a carbon atom is 0.2 nm, and distances of 0.3–0.4 nm favor the formation of van der Waals interactions between two carbon atoms. According to the distances between INT and residues around the pocket shown in Fig. 5, we speculate that van der Waals interactions provide the major contribution to the bonding of INT and HA. This speculation was verified as described in the next section.

### Verification of binding mode of INT-HA under neutral conditions

**Figure 6:**
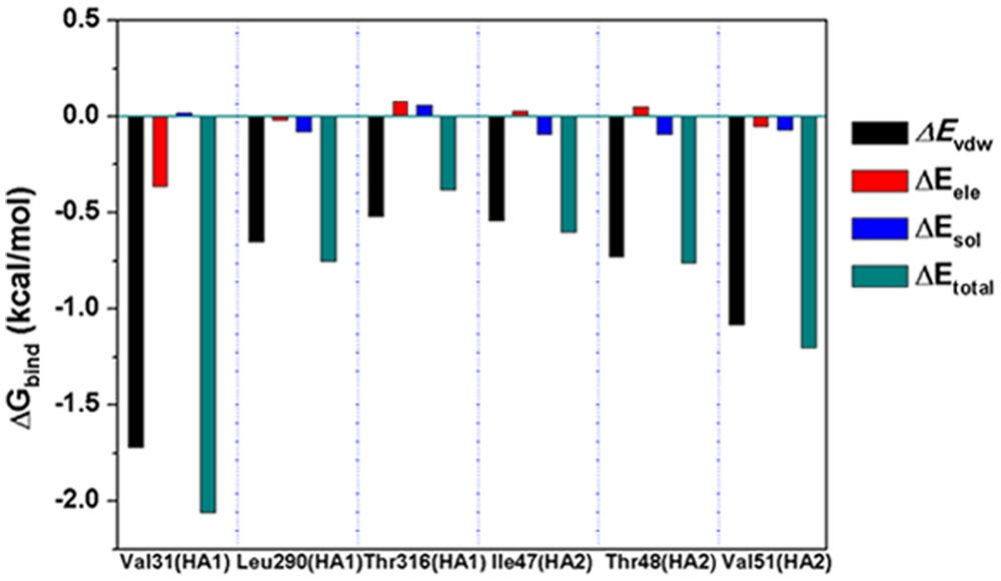
Decomposition of the binding energy on per residue basis on INT-HA under neutral conditions.

In order to further validate the binding modes and obtain detailed information on the key residues of the binding pocket in the complex, the electrostatic, van der Waals, solvation and total contribution of the residues to the binding free energy of the INT-HA complex were calculated with the MM-PBSA method. To achieve more accurate results, MM-PBSA calculations were carried out twice. The snaps used in calculations were respectively extracted from equilibrium stages within first and second 100 ns. By comparing the energy of the two results, similarity could be observed (Fig. S6), thus, only one group of data from the equilibrium stages within the first 100 ns was shown in this section. The contributions from the residues around INT are shown in Fig. 6 and Table 2.

**Table 2:** Decomposition of binding energy on per residue basis on INT-HA under neutral conditions.

Residue	*ΔE*_*vdw*_ (kcal mol^−1^)	*ΔE*_*ele*_ (kcal mol^−1^)	*ΔE*_*sol*_ (kcal mol^−1^)	*ΔE*_*total*_ (kcal mol^−1^)
Val31(HA1)	−1.72	−0.36	0.02	−2.06
Leu290(HA1)	−0.65	−0.02	−0.08	−0.75
Thr316(HA1)	−0.52	0.08	0.06	−0.38
Ile47(HA2)	−0.54	0.03	−0.09	-0.60
Thr48(HA2)	−0.73	0.05	−0.09	−0.76
Val51(HA2)	−1.08	−0.05	−0.07	−1.2

The contribution of selected residues surrounding the INT-HA complex binding pocket can be clearly seen from Fig. 6 and Table 2. As shown, HA1-Val31, HA1-Leu290, HA1-Thr316, HA2-Ile47, HA2-Thr48 and HA2-Val51 have favorable total energy contributions, suggesting that these residues participate in the complex binding. More importantly, for aforementioned residues van der Waals interactions play a major role in the total energy contribution as evidenced by lower values of *ΔE*_*vdw*_ (−1.72, −0.65, −0.52, −0.54, −0.73 and −1.08 kcal·mol^−1^, respectively). The free energy calculation results are consistent with the speculation above that van der Waals interactions are a major contributor to the bonding of INT and HA.

The total binding energy of the INT-HA complex systems, an important standard measure of binding affinity between INT and HA, were calculated as well. Total binding energies of INT-HA_Washington_ and INT-HA_Florida_ were calculated by MM-PBSA at the same time. As listed in Table 3, the results show that the values of *ΔG*_*total*_ for INT-HA_Washington_ and INT-HA_Florida_ are −16.15 and −16.02 kcal·mol^−1^, respectively, indicating that INT has similar binding interactions with HA_Washington_ and HA_Florida_. The calculated result is qualitatively consistent with the experimental results showing that INT could inhibit HA of A/Washington/10/2008(H1N1) and A/Florida/21/2008(H1N1) at similar levels (IC_50_ of 0.29 µM and 0.3 µM) *in vitro* ^16^.

**Table 3:** Calculation of binding free energy using MM/PBSA under neutral conditions.

Energy components (kcal mol^−1^)	INT-HA_Washington_	INT-HA_Florida_
*Δ*E_ele_	−1.04	0.19
*Δ*E_vdw_	−18.29	−19.42
*Δ*G_PB_	6.84	6.54
*Δ*G_np_	−3.66	−3.33
Nonpolar	−21.95	−22.75
Polar	5.80	6.73
*Δ*G_total_	−16.15	−16.02

Contributions from total free energy were analyzed in detail as well. The *ΔG*_*total*_ can be divided into polar and nonpolar energies. The free energies of INT binding to two HAs were found to be primarily derived from the nonpolar energies (equal to *ΔG*_*np*_ + *ΔE*_*vdw*_), which were −21.95 kcal·mol^−1^ in the INT-HA_Washington_ complex and −22.75 kcal·mol^−1^ in the INT-HA_Florida_ complex. However, for both complexes, the polar energy (equal to *ΔG*_*PB*_ + *ΔE*_*ele*_) showed negative contributions at 5.80 and 6.73 kcal·mol^−1^ for INT-HA_Washington_ and INT-HA_Florida_, respectively. In addition, the values of *ΔE*_*vdw*_ for INT-HA_Washington_ and INT-HA_Florida_ were calculated at −18.29 and −19.42 kcal·mol^−1^, respectively, indicating that van der Waals interactions are dominant in the total energy contribution. The total free energy calculations also were in agreement with the speculation above, indicating that nonpolar interactions, especially van der Waals interactions, are the major contributor to the bonding of INT and HA.

Thus, the above analysis suggests that INT can stably bind with HA under neutral conditions via van der Waals interactions and has the ability to enter the endosome following HA binding during endocytosis.

### Binding and dynamics behavior of INT at low pH conditions

In the above discussion, effective binding of INT with HA under neutral conditions was demonstrated. Nevertheless, the ability of INT to maintain the interaction with HA at a low pH is crucial to its inhibition and determines whether it has the potential to be a HA entry inhibitor. Prior experimental data indicated that INT indeed could inhibit different influenza virus H1N1 strains *in vitro* ^16^, but its mechanism of inhibition at low pH at the atom level has not been explored. In this section, the binding and dynamics behavior of INT at low-pH conditions will be discussed.

As described in method, HA_1ruz_ was chosen as the low-pH model in this section based on the calculated pK_a_ of the titratable residues by Zhou *et al*. In order to determine whether the INT binding would affect the protonation state of the residues, the titratable residues’ locations of HA_1ruz_ were investigated. Most titratable residues were located far away from the INT binding site except HA1-Lys278, HA1-Glu302 and HA2-Glu56. Subsequently, the pKa of the above three residues were calculated via PROPKA^45, 46^ with or without INT (Table S2). It’s further illustrated that the bound of INT could not affect the protonation state of the residues. Thus, protonation state of HA_1ruz_ could be assigned according to the results of Zhou *et al*. directly (Table S1).

**Figure 7:**
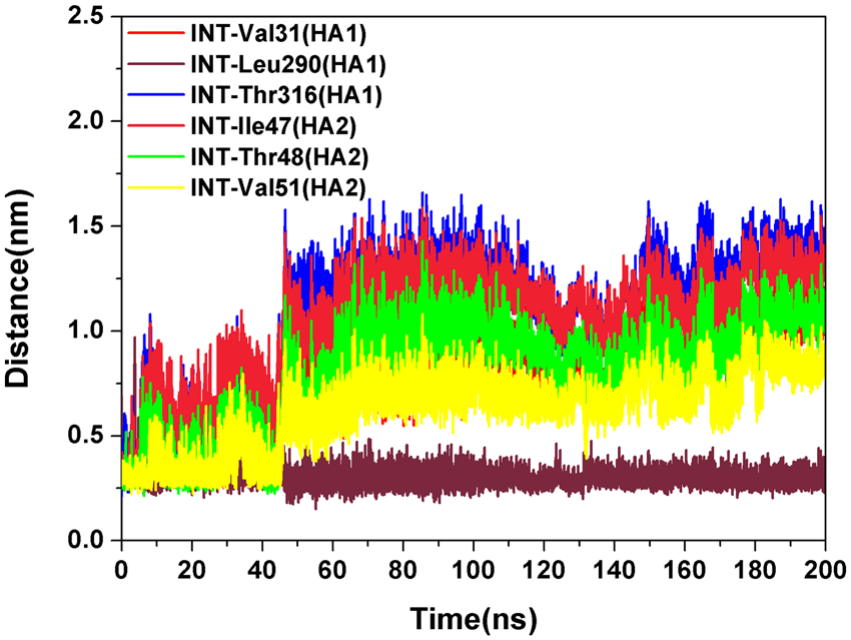
Distances between INT and residues (HA1-Val31, HA1-Leu290, HA1-Thr316, HA2-Ile47, HA2-Thr48 and HA2-Val51) under acidic conditions.

In order to facilitate the comparison, for the investigated complex in this study only one monomer of the HA trimer was bound with INT, and the other two unbound monomers still could disaggregate at low pH as usual. In order to monitor the location of INT at low-pH conditions, distances between INT and its binding residues (HA1-Val31, HA1-Leu290, HA1-Thr316, HA2-Ile47, HA2-Thr48 and HA2-Val51) in the neutral state were calculated again. As shown in Fig. 7, almost all the distances between INT and its binding residues (in neutral state) were disrupted at different degrees at 0–45 ns and increased sharply at about 46 ns, except for HA1-Leu290. The disrupted distances kept at about 65–200 ns with small fluctuation. These observations suggest that INT had relocated out of the pocket formed by the side chain of HA1-Val31, HA1-Leu290, HA1-Thr316, HA2-Ile47, HA2-Thr48 and HA2-Val51 and moved to a new low potential energy point at 65–200 ns. The distance between INT and Leu290-HA1 was always maintained at about 0.3–0.4 nm, which indicated that the new binding location was still nearby. In order to confirm this phenomenon, an independent repeated trial was conducted with a different computing resource with the same parameters, and the same results were obtained (Fig. S7). Therefore, we suspected that the binding of INT with HA under neutral conditions only facilitates its intake, and another mechanism exists for the INT binding and inhibition of HA at low-pH conditions.

**Figure 8:**
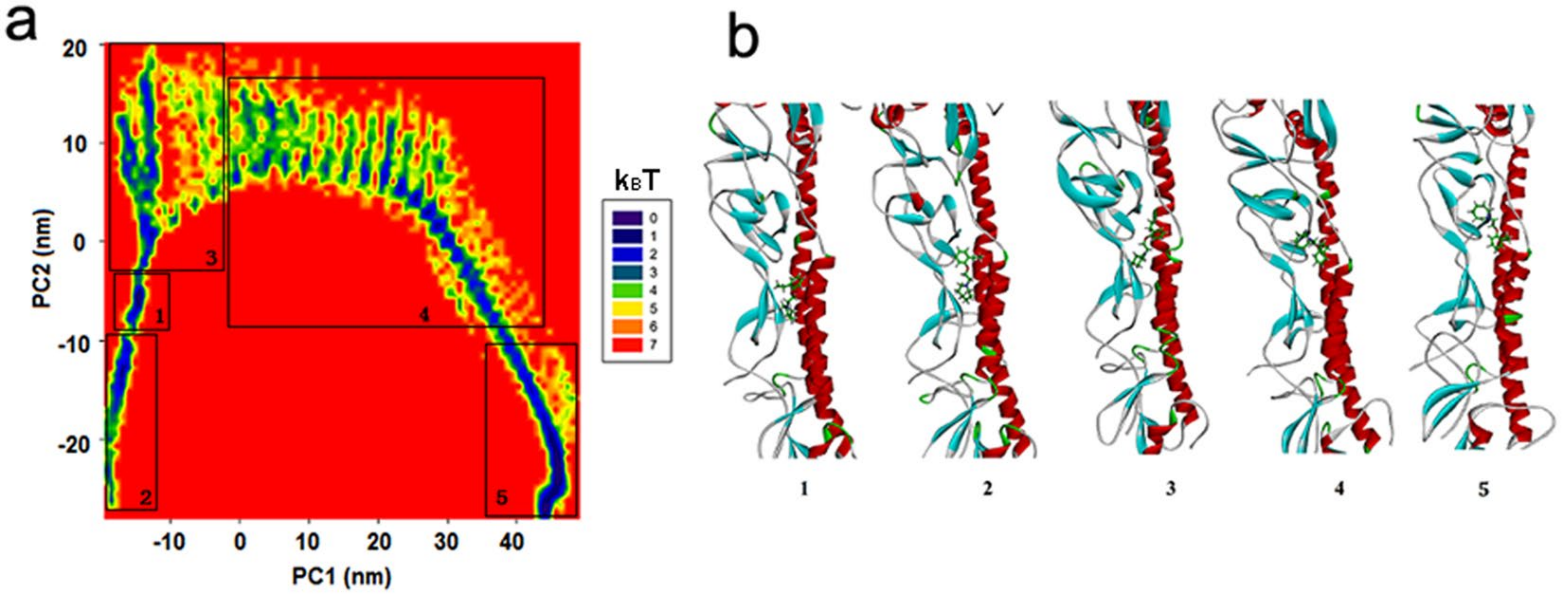
(**a**) Relative free energy surfaces along the first two principle components (PC-1, PC-2) of INT-HA_1ruz_ under acidic conditions. (**b**) Five representative conformations extracted from different areas.

In order to explore the binding and dynamic behavior of INT at low pH, a cluster analysis of INT-HA_1ruz_ was performed. As shown in Fig. 8a, unlike with INT-HA under neutral conditions (Fig. 4a), mainly five blue areas could be found in the free energy surface plot of the complex at low pH. Most conformations of 1–8 ns, 9–28 ns, 29–64 ns, 65–145 ns and 146–200 ns were clustered in regions 1, 2, 3, 4 and 5, respectively. Five representative conformations extracted from different areas are shown in Fig. 8b. After analysis, INT could bind with HA in at least four dominant positions at low-pH conditions. In the initial stage, INT bound between HA1 and the short helix of HA2, but INT moved to the HA head region gradually over time. According to the cluster analysis, INT kept the fourth binding state (Fig. 8b (4 and 5)) with HA after 65 ns, which is in agreement with the results from Fig. 7.

Due to the bulky head region of HA1 being rich in protonated residues at low pH, a large repulsion promoted the structural disaggregation at the initial stage of the simulation^13, 14^. As the initial binding site of INT was far away from the positively charged head, INT could not prevent the disaggregation at the initial stage. We propose that following the disaggregation of HA1 and the shift of charged state of HA2, a new binding site might be revealed and thus enabled the purposeful relocation of INT^46^.

**Figure 9:**
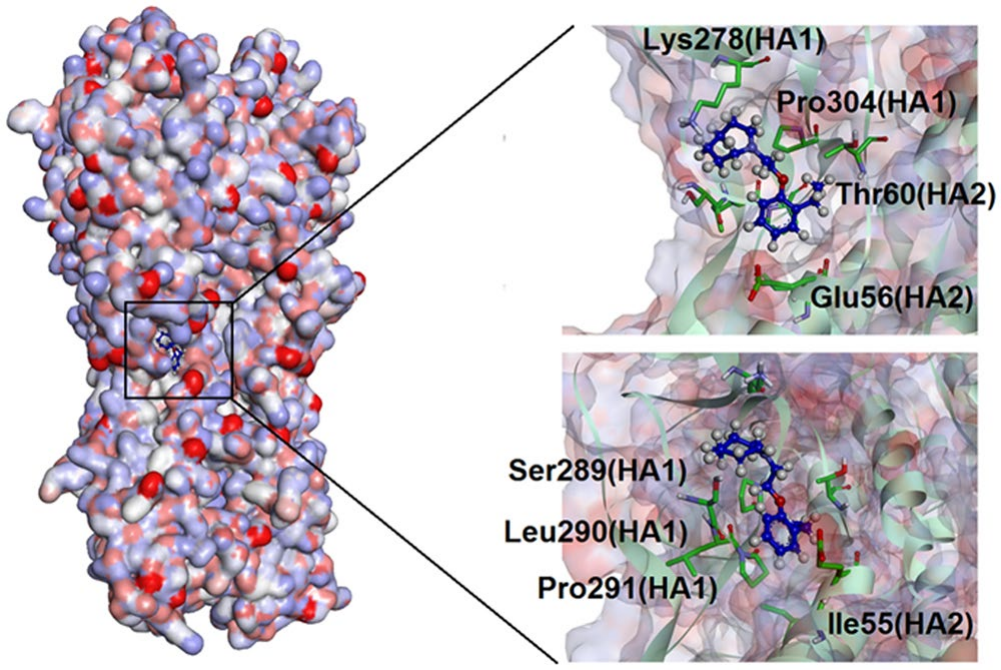
Predicted new binding modes of INT-HA_1ruz_ under acidic conditions.

The new detailed binding mode of the INT-HA_1ruz_ complex at low pH was investigated and verified by simulation and MM-PBSA calculation twice as described above (Fig. S8). The relevant results are provided in Fig. 9 and Table 4. As shown, a new pocket bound by INT at low pH was found to be surrounded by HA1-Lys278, HA1-Ser289, HA1-Leu290, HA1-Pro291, HA1-Pro304, HA2-Ile55, HA2-Glu56 and HA2-Thr60. In detail, the seven-member heterocycle of INT could locate between side chains of HA1-Lys278 and HA1-Ser289. Side chain of HA1-Pro304 is close to C9 of INT and the side chain of HA2-Thr60 is adjacent to C8 of INT. HA1-Leu290, HA1-Pro291, HA2-Ile55 and HA2-Glu56 together are approaching the six-member ring of INT. The exposed new binding site is still located on the interface between HA1 and HA2. According to the data calculated by MM-PBSA, van der Waals interactions were confirmed to play a major role in the total energy contribution for aforementioned residues as well as evidenced by lower values of *ΔE*_*vdw*_ (−1.07, −1.58, −1.72, −2.03, −1.43, −0.58, −1.03 and −0.98 kcal·mol^−1^, respectively) as shown in Table 4. Although there are four polar residues, HA1-Lys278, HA1-Ser289, HA2-Glu56 and HA2-Thr60, locating around the new binding site, the polarity of these residues still could not contribute to INT binding due to the molecular property of INT. Furthermore, all the polar part of the side chains from the above residues were redirected to the outside of the binding pockets. The sequence conservation of eight residues around the new binding site was evaluated by sequence alignment of the different H1N1 influenza virus strains in the recent 30-year period of time. The results (Table 5) indicated that all the investigated residues in the new binding site are highly conserved, implying that the new binding pocket has the potential to be an effective drug target.

**Table 4:** Decomposition of binding energy on per residue in new binding site basis on INT-HA_1ruz_ under acidic conditions.

Residue	*ΔE*_*vdw*_ (kcal mol^−1^)	*ΔE*_*ele*_ (kcal mol^−1^)	*ΔE*_*sol*_ (kcal mol^−1^)	*ΔE*_*total*_ (kcal mol^−1^)
Lys278(HA1)	−1.07	−0.63	0.72	−0.98
Ser289(HA1)	−1.58	0.06	0.91	−0.62
Leu290(HA1)	−1.72	−0.78	1.05	−1.45
Pro291(HA1)	−2.03	−0.11	0.34	−1.80
Pro304(HA1)	−1.43	0.14	−0.10	−1.39
Ile55(HA2)	−0.58	−0.28	0.47	−0.39
Glu56(HA2)	−1.03	0.35	−0.11	−0.79
Thr60(HA2)	−0.98	−0.07	0	−1.06

**Table 5:** Sequence conservation of residues around new binding site evaluated by sequence alignment for different H1N1 influenza virus strains in recent 30-year period of time.

Residue	Entropy data*	Residue	Entropy data
Lys278(HA1)	0.42889	Pro304(HA1)	0.06495
Ser289(HA1)	0.04048	Ile55(HA2)	0.05048
Leu290(HA1)	0.03910	Glu56(HA2)	0.05353
Pro291(HA1)	0.03809	Thr60(HA2)	0.05977

*low entropy data means high conservation.

### Mechanism of INT Inhibiting HA disaggregation at low pH conditions

Based on analysis of its amino acid sequence, the HA2 polypeptide was found to contain the well-known heptad-repeat module of hydrophobic/hydrophilic amino-acids, which is crucial for the formation of coiled-coil structures. In order to drive the loop-to-helix transition and the formation of a coiled-coil structure, it is necessary for water molecules to directly interact with the hydrophilic residues^15^. Therefore, the ability of INT to stabilize the connection between HA1 and HA2 and to effectively prevent water from entering into the interior of HA2 is crucial for the inhibitory activity of INT. Because INT is located on the interface between HA1 and HA2, it is considered to not only be able to stabilize HA1 and HA2 but also prevent the entry of water into the interior of HA and further influence the rearrangement of HA2.

**Figure 10:**
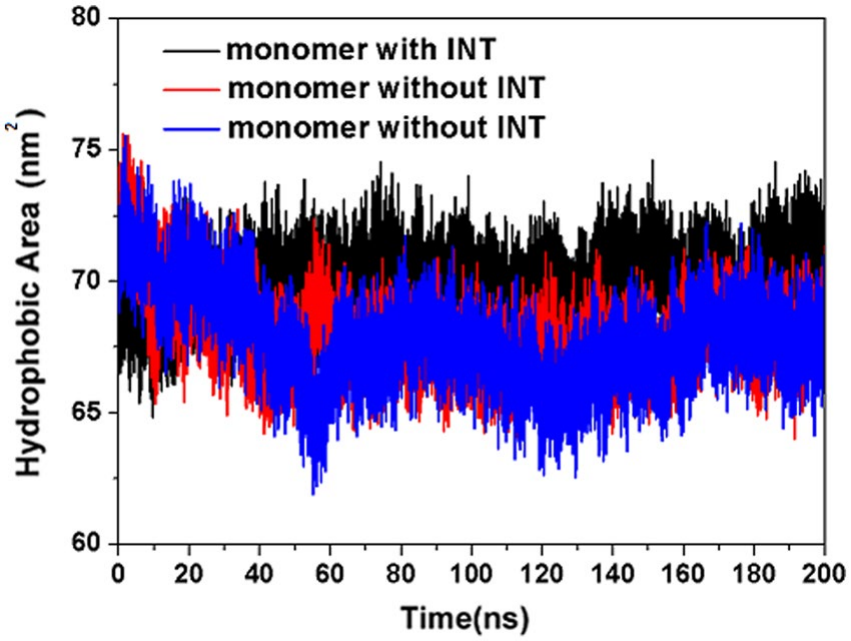
Hydrophobic areas of HA2 subunits with or without INT during 200 ns molecular dynamics simulations under acidic conditions.

In order to investigate the different degrees of water accessibility, the hydrophobic areas of HA2 subunits with or without INT were monitored during 200 ns dynamics simulation at low pH. As shown in Fig. 10, the hydrophobic area of HA2 subunits bound with INT always stabilized at about 70 nm^2^, but those of the other two free monomers (HA monomers bound without INT) clearly decreased at a value below 70 nm^2^ as the simulation. The reduced hydrophobic area of the free HA2 subunit indicated that the solution gained access to the inside of the protein due to the lack of protection by INT. By contrast, the HA2 subunit from monomers bound with INT consistently retained the larger hydrophobic area throughout the simulation.

**Figure 11:**
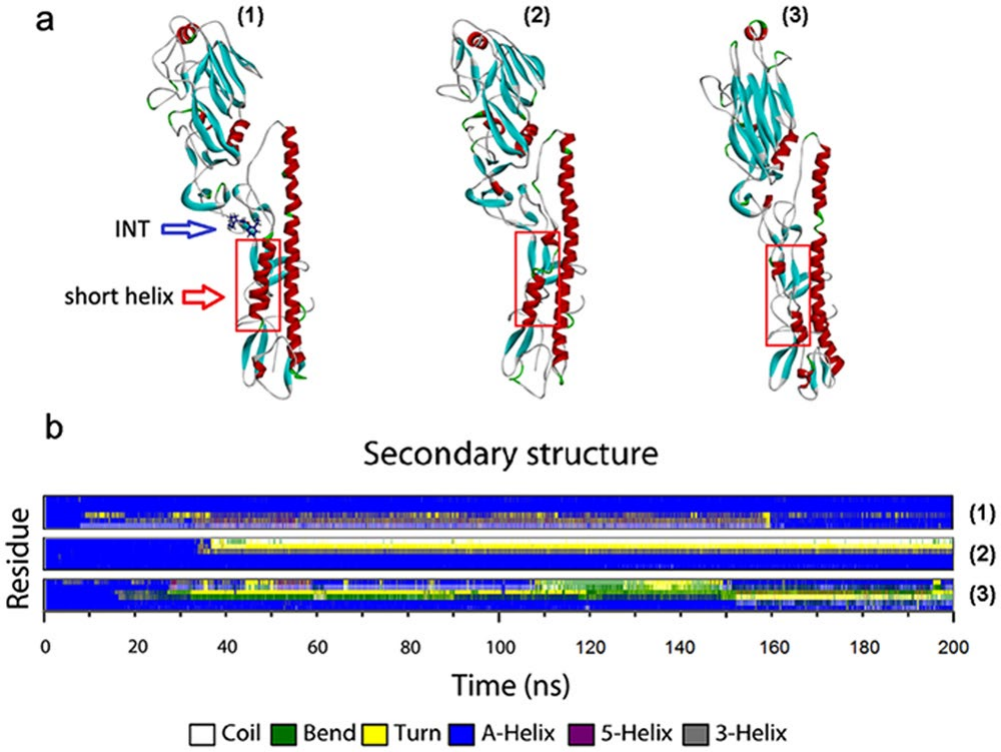
(**a**) Differences in structure between HA bound with INT and free HA under acidic conditions. (**b**) Differences of secondary structures of short helixes between HA bound with INT and free HA during 200 ns molecular dynamics simulations under acidic conditions.

**Figure 12:**
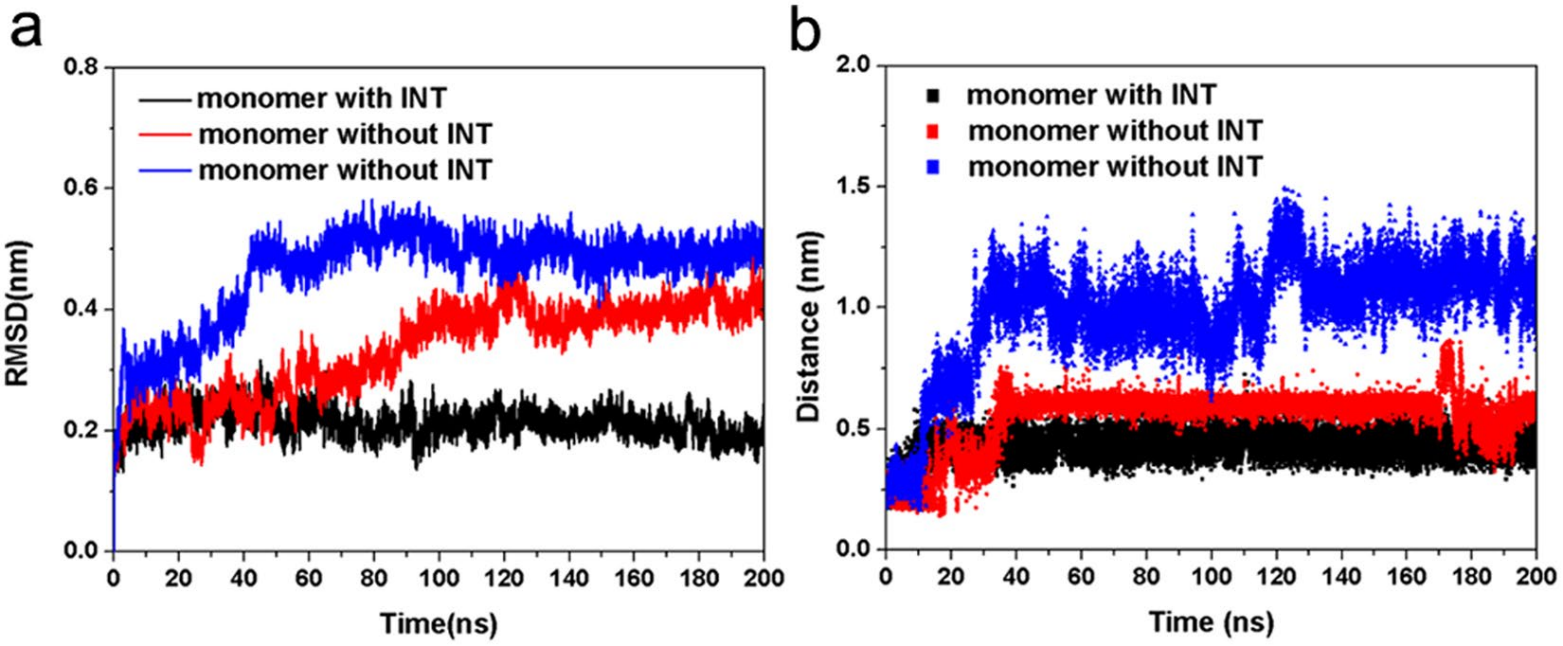
(**a**) RMSD plot of loop from HA bound with INT and free HA during 200 ns molecular dynamics simulations under acidic conditions. (**b**) Changes in distances of Glu101 and Lys50 during 200 ns molecular dynamics simulations under acidic conditions.

A helix-loop-helix structure provides stability for HA2, and the entry of water must lead to a structural change of this molecule to some extent. We investigated the difference between HA bound with INT and free HAs. As shown in Fig. 11a(1), due to the bound INT, HA preferably maintained the initial helix-loop-helix structure, while the short helixes of the two free HA clearly changed. The secondary structures of the three short helixes were monitored during the 200 ns simulation. The command “do_dssp” of Gromacs was used for secondary structure calculation. As shown in Fig. 11b, the great structural fluctuations in the short helixes of free HAs could be observed at about 30 ns, and the distorted short helixes could endure till 200 ns. These distortions were mainly composed of the formations of the coil, bend and turn during the conformation simulation. By contrast, it’s found that the corresponding secondary structure of HA could display much more stability in the presence of INT. Except for the influence of INT on the short helix, we suspected that the binding of INT also could maintain the stability of the loops connecting the short and long helices. The stability of loops from each HA2 subunit was measured by the RMSD of loops. As shown in Fig. 12a, the RMSD of the loop from the monomer bound with INT was very stable and held the value at about 0.2 nm, indicating that the binding of INT could maintain the rigidity of the loop. RMSD of loops from the two free monomers fluctuated more obviously, which indicated a disruption in the rigidity of the loops. Differences in the loops can be observed in Fig. 11a as well.

In addition, the side chain dynamic behavior of a group of reported residues (Glu101 and Lys50) was also monitored. The salt bridge between Glu101 and Lys50 is crucial for the stabilization of short and long helices of HA2, and the break of this salt bridge would promote the loop-helix transformation^27^. Therefore, stabilization in the distance between these two residues is an important measure of protective efficacy of INT. From the results shown in Fig. 12b, the distances between Glu101 and Lys50 in the three monomers were found to be different. In the two free monomers, this distance was clearly increased and not favorable for keeping the salt bridge. By contrast, the corresponding distance in the monomer bound with INT was always stable at a lower value, which indicated that the salt bridge of this monomer was protected well and the helix-loop-helix was more stable.

According to the above analysis, we may conclude that INT has the potential to effectively prevent water from entering into the interior of HA, maintain the rigidity of the HA2 loop and stabilize the distance between long helix and short helix, and further obstruct the loop-to-helix transition.

## Conclusion

In the current study, the mechanism of binding and inhibition of INT, a potential influenza virus HA inhibitor, was explored by molecular dynamics simulation and MM-PBSA calculation. The behaviors of INT under neutral and acidic conditions were investigated at the same time, and an interesting dynamic binding mechanism of INT was found. We propose that the binding of INT with HA under neutral conditions allows for its intake, while the interaction of INT with HA at low-pH conditions at a new binding site employs a different mechanism to carry out its inhibitory effect. After a series of confirmation, we believe that the binding of INT can prevent HA1 from disassociating from HA2, further maintain the rigidity of the HA2 loop and stabilize the distance between the long helix and short helix.

The investigated residues in the new binding site are highly conserved, implying that the new binding pocket has the potential to be an effective drug target. The results in this study will be useful for the development of efficient drug targets and provide a theoretical basis for the mechanism of new influenza virus inhibitors.

## Electronic supplementary material

Supplementary Information Files
