# Supplementary material for: Exploration of binding and inhibition mechanism of a small molecule inhibitor of influenza virus H1N1 hemagglutinin by molecular dynamics simulation
Source: Sci Rep. 2017 Jun 19;7:3786. doi: 10.1038/s41598-017-03719-4 (PMC5476670; doi:10.1038/s41598-017-03719-4)
Supplement: Supplementary file 1 — Supplementary Information Files [file 41598_2017_3719_MOESM1_ESM.pdf]

Exploration of binding and inhibition mechanism of a small molecule inhibitor of influenza virus H1N1 hemagglutinin by molecular dynamics simulation

Shanshan Guan[a], Tianao Wang[a,d], Ziyu Kuai[a], Mengdan Qian[c], Xiaopian Tian[a], Xiuqi Zhang[a], Yongjiao Yu[a], Song Wang[c*], Hao Zhang[c], Hao Li[d], Wei Kong[a,b], Yaming Shan[a,b*].

[a] National Engineering Laboratory for AIDS Vaccine, School of Life Sciences, Jilin University, Changchun, Jilin, China

[b] Key Laboratory for Molecular Enzymology and Engineering, The Ministry of Education, School of Life Sciences, Jilin University, Changchun, Jilin, China

[c] Laboratory of Theoretical and Computational Chemistry, Institute of Theoretical Chemistry, Jilin University, Changchun, Jilin, China

[d] College of Biology and Food Engineering, Jilin Engineering Normal University, Changchun, Jilin, China

[*]Correspondence to: Yaming Shan, Song Wang

E-mail: shanym@jlu.edu.cn; ws@jlu.edu.cn.

Telephone: 0086-431-89228979

Fax: 0086-431-85167751

a

```
        10        20        30        40        50        60        70        80        90
SDTLCIGYHANNSTDTVDTVLEKNVTVTHSVNLLEDKHNGKLCKLRGVAPLHLGKCNIAGWILGNPECESLSTASSWSYIVETSSSDNGT
ADTICIGYHANNSTDTVDTVLEKNVTVTHSVNLLEDNHNGKLCLLKGIAPLQLGNCSVAGWILGNPECELLISKESWSYIVEKPNPENGT
ADTICIGYHANNSTDTVDTVLEKNVTVTHSVNLLENSHNGKLCLLKGIAPLQLGNCSVAGWILGNPECELLISKESWSYIVEKPNPENGT

       100       110       120       130       140       150       160       170       180
CYPGDFIDYEELREQLSSVSSFERFEIFPKTSSWPNHDSNKGVTAACPHAGAKGFYKNLIWLVKKGNSYPKLSKSYINDKGKEVLVLWGI
CYPGHFADYEELREQLSSVSSFKRFEIFPKESSWPNHTVT-GVSASCSHNGESSFYKNLLWLTGKNGLYPNLSKSYANNKEKEVLVLWGV
CYPGHFADYEELREQLSSVSSFERFEIFPKESSWPNHTVT-GVSASCSHNGESSFYRNLLWLTGKNGLYPTLSKSYANNKEKEVLVLWGV

       190       200       210       220       230       240       250       260       270
HHPSTTADQQSLYQNADTYVFVGTSRYSKKFKPEIAIRPKVRDQEGRMNYYWTLVEPGDKITFEATGNLVVPRYAFAMERNAGSGIIISD
HHPPNIGNQMTLYHKENAYVSVVSSHYSRKFTPEIAKRPKVRDQEGRINYYWTLLEPGDTIIFEASGNLIAPRYAFALSRGFGSGIINSN
HHPPNIVDQKTLYRTENAYVSVVSSHYSRKFTPEIAKRPKVRDQEGRINYYWTLLEPGDTIIFEANGNLIAPRYAFALSRGFGSGIINSN

       280       290       300       310       320       330       340       350       360
TPVHDCNTTCQTPKGAINTSLPFQNIHPITIGKCPKYVKSTKLRLATGLRNVP-LFGAIAGFIEGGWTGMVDGWYGYHHQNEQGSGYAAD
APMDECDAKCQTPQGAINSSLPFQNVHPVTIGECPKYVRSAKLRMVTGLRNIP-LFGAIAGFIEGGWTGMVDGWYGYHHQNEQGSGYAAD
APMDKCDAKCQTPQGAINSSLPFQNVHPVTIGECPKYVRSAKLRMVTGLRNIP-LFGAIAGFIEGGWTGMVDGWYGYHHQNEQGSGYAAD

       370       380       390       400       410       420       430       440       450
LKSTQNAIDKITNKVNSVIEKMNTQFTAVGKEFNHLEKRIENLNKKVDDGFLDIWTYNAELLVLLENERTLDYHDSNVKNLYEKVRNQLK
QKSTQNAINGITNKVNSVIEKMNTQFTAV-KEFNKLERRMENLNKKVDDGFIDVWTYNAELLVLLENERTLDFHDSNVKNLYEKVKNQLK
QKSTQNAINGITNKVNSVIEKMNTQFTAVGKEFNKLERRMENLNKKVDDGFIDIWTYNAELLVLLENERTLDFHDSNVKBLYEKVKSQLK

       460       470       480       490
NNAKEIGNGCFEFYHKCDNTCMESVKNGTYDYPKYSEEAKLNREE
NNAKEIGNGCFEFYHKCNDECMESVKNGTYDYPKYSEESKLSREK
NNAKEIGNGCFEFYHKCNDECMESVKNGTYDYPKYSEESKLNKEK
```

b

```
        10        20        30        40        50        60        70        80        90
ADTICIGYHANNSTDTVDTVLEKNVTVTHSVNLLEDNHNGKLCLLKGIAPLQLGNCSVAGWILGNPECELLISKESWSYIVEKPNPENGT
ADTICIGYHANNSTDTVDTVLEKNVTVTHSVNLLENSHNGKLCLLKGIAPLQLGNCSVAGWILGNPECELLISKESWSYIVEKPNPENGT
ADTICIGYHANNSTDTVDTVLEKNVTVTHSVNLLEDSHNGKLCKLKGIAPLQLGKCNIAGWLLGNPECDLLLTASSWSYIVETSNSENGT

       100       110       120       130       140       150       160       170       180
CYPGHFADYEELREQLSSVSSFKRFEIFPKESSWPNHTVT-GVSASCSHNGESSFYKNLLWLTGKNGLYPNLSKSYANNKEKEVLVLWGV
CYPGHFADYEELREQLSSVSSFERFEIFPKESSWPNHTVT-GVSASCSHNGESSFYRNLLWLTGKNGLYPTLSKSYANNKEKEVLVLWGV
CYPGDFIDYEELREQLSSVSSFEKFEIFPKTSSWPNHETTKGVTAACSYAGASSFYRNLLWLTKKGSSYPKLSKSYVNNKGKEVLVLWGV

       190       200       210       220       230       240       250       260       270
HHPPNIGNQMTLYHKENAYVSVVSSHYSRKFTPEIAKRPKVRDQEGRINYYWTLLEPGDTIIFEASGNLIAPRYAFALSRGFGSGIINSN
HHPPNIVDQKTLYRTENAYVSVVSSHYSRKFTPEIAKRPKVRDQEGRINYYWTLLEPGDTIIFEANGNLIAPRYAFALSRGFGSGIINSN
HHPPTGTDQQSLYQNADAYVSVGSSKYNRRFTPEIAARPKVRDQAGRMNYYWTLLEPGDTITFEATGNLIAPWYAFALNRGSGSGIITSD

       280       290       300       310       320       330       340       350       360
APMDECDAKCQTPQGAINSSLPFQNVHPVTIGECPKYVRSAKLRMVTGLRNIP----LFGAIAGFIEGGWTGMVDGWYGYHHQNEQGSGY
APMDKCDAKCQTPQGAINSSLPFQNVHPVTIGECPKYVRSAKLRMVTGLRNIP----LFGAIAGFIEGGWTGMVDGWYGYHHQNEQGSGY
APVHDCNTKCQTPHGAINSSLPFQNIHPVTIGECPKYVRSTKLRMATGLRNIPAR-GLFGAIAGFIEGGWTGMIDGWYGYHHQNEQGSGY

       370       380       390       400       410       420       430       440       450
AADQKSTQNAINGITNKVNSVIEKMNTQFTAV-KEFNKLERRMENLNKKVDDGFIDVWTYNAELLVLLENERTLDFHDSNVKNLYEKVKN
AADQKSTQNAINGITNKVNSVIEKMNTQFTAVGKEFNKLERRMENLNKKVDDGFIDIWTYNAELLVLLENERTLDFHDSNVKBLYEKVKS
AADQKSTQNAIDGITNKVNSVIEKMNTQFTAVGKEFNNLERRIENLNKKVDDGFLDIWTYNAELLVLLENERTLDFHDSNVRNLYEKVKS

       460       470       480       490
QLKNNAKEIGNGCFEFYHKCNDECMESVKNGTYDYPKYSEESKLSREK
QLKNNAKEIGNGCFEFYHKCNDECMESVKNGTYDYPKYSEESKLNKEK
QLKNNAKEIGNGCFEFYHKCDDACMESVRNGTYDYP------------
```

Figure S1 (a) Sequence alignment results between template (PDB ID: 4LXV) and

preferred models (HA from H1N1 Washington and Florida strains). 1st line: 4LXV, 2nd line: HA from H1N1 Washington strains, 3rd line: HA from H1N1 Florida strains. (b) Sequence alignment results between 1RUZ and preferred models (HA from H1N1 Washington and Florida strains). 1st line: HA from H1N1 Washington strains, 2nd line: HA from H1N1 Florida strains, 3rd line: 1RUZ.

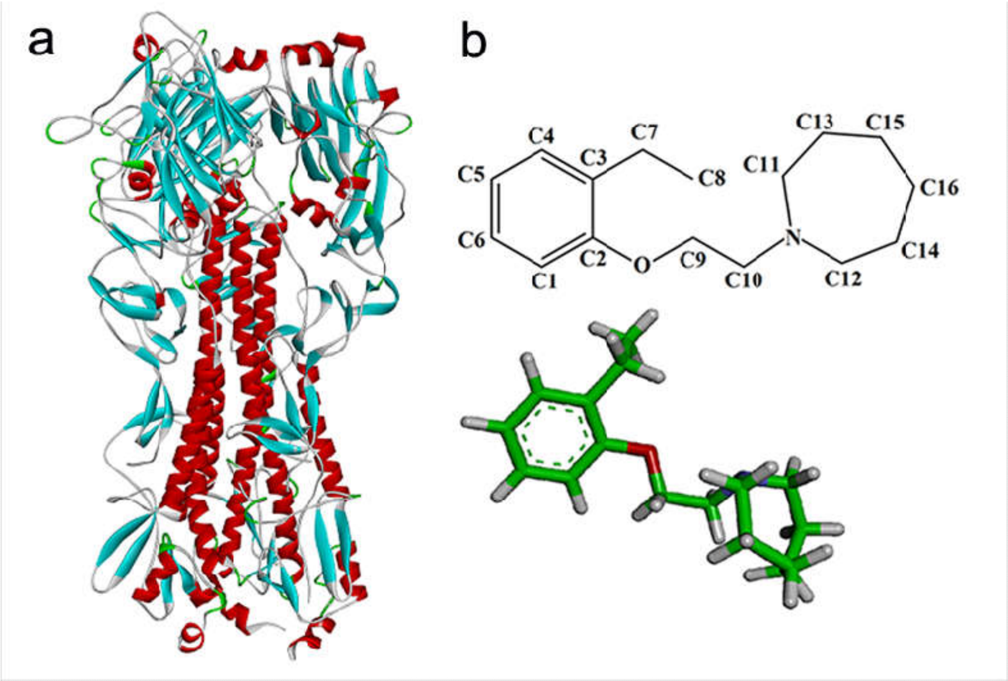

Figure S2 (a) Structure of HA. (b) Structure of INT.

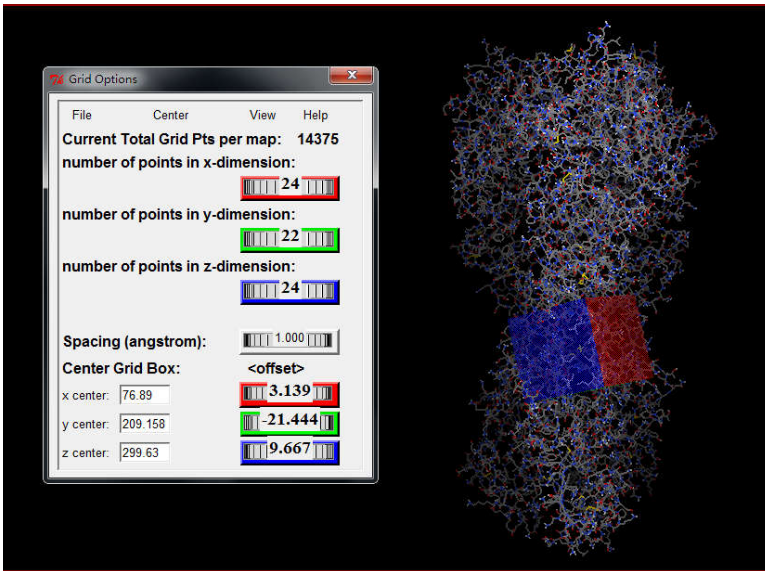

Figure S3 Docking grid for HA.

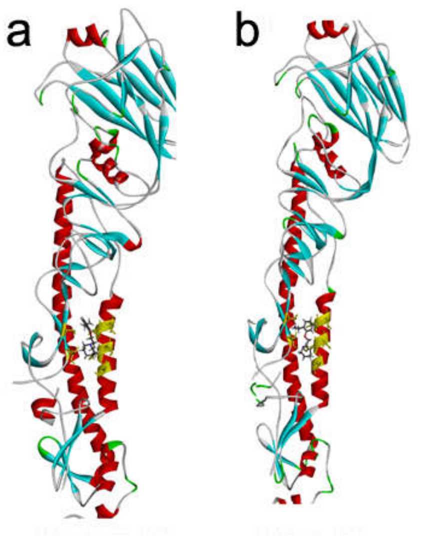

Figure S4 Docking results of INT-HA. (a) INT-$HA_{Washington}$. (b) INT-$HA_{Florida}$.

Comparisons of docking results with previous results from Arnab *et al.*.

Arnab *et al.* presented a molecular dynamics model of the complex that consisted of INT and H5 HA which was another member of Group 1 HA. In their prediction, INT was located at the binding site which be surrounded by HA1-T318, HA1-Q40, HA2-I45, HA2-V48, HA2-T49, HA2-V52 and HA2-N53 (number in H5 HA). In addition, their results indicated that the mutations at residues HA2-Ile45, HA2-Val52, and HA2-Asn53 could greatly reduce the potency of the compound, which revealed the position of INT bound. It is well known that, the conservative stems from H1 HA and H5 HA are highly homologous. The residues mentioned above, HA1-T318, HA2-I45, HA2-V48, HA2-T49, HA2-V52 and HA2-N53 (number in H5 HA) in their work were corresponded respectively to HA1-T316, HA2-I44, HA2-I47, HA2-T48, HA2-V51 and HA2-N52 (number in H1 HA) in our study. The binding site we predicted could be in quite accordance with their mutation and theoretical docking experiments, which indicated that our docking results could be applied to the next molecular dynamics study.

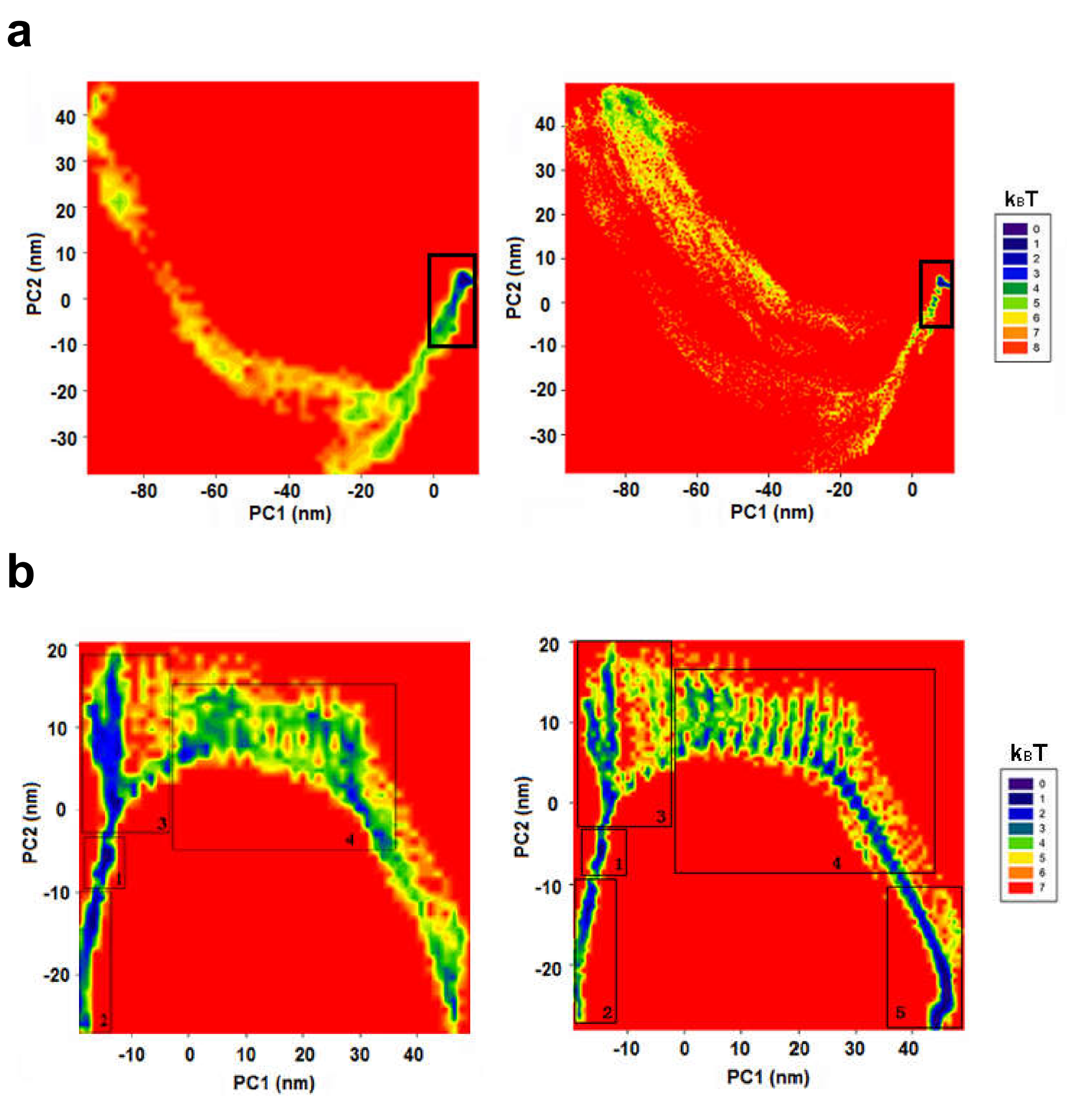

Figure S5 Comparison plots between the free energy surface with the first 100 ns and further one with the extended 100 ns in complexes (a) INT-HA$_{\text{Washington}}$ (b) INT-HA$_{\text{1RUZ}}$.

Cluster position of INT-HA$_{\text{Washington}}$ has not changed much during extended simulation as shown in (a). For INT-HA$_{\text{1ruz}}$ (acid condition), new blue region (region 5) could be found in the right plat of (b) as expected. With the increasing time of simulation under acid condition, structures of the two free monomers (without INT bound) would have been kept changing continually, which could trigger the new region (region 5) appeared in the right plat of (b). Nevertheless, INT was still located at the

new predicted binding site in the conformations extracted from region 5 (Fig. 8b) indicating the stability of INT bound-monomer. The similarities among free energy surfaces further indicated the convergence of systerms.

a

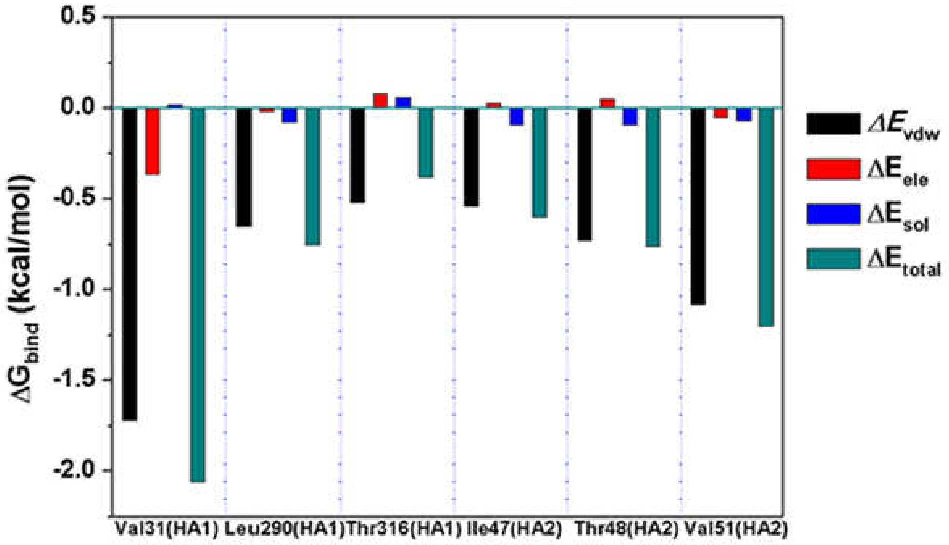

b

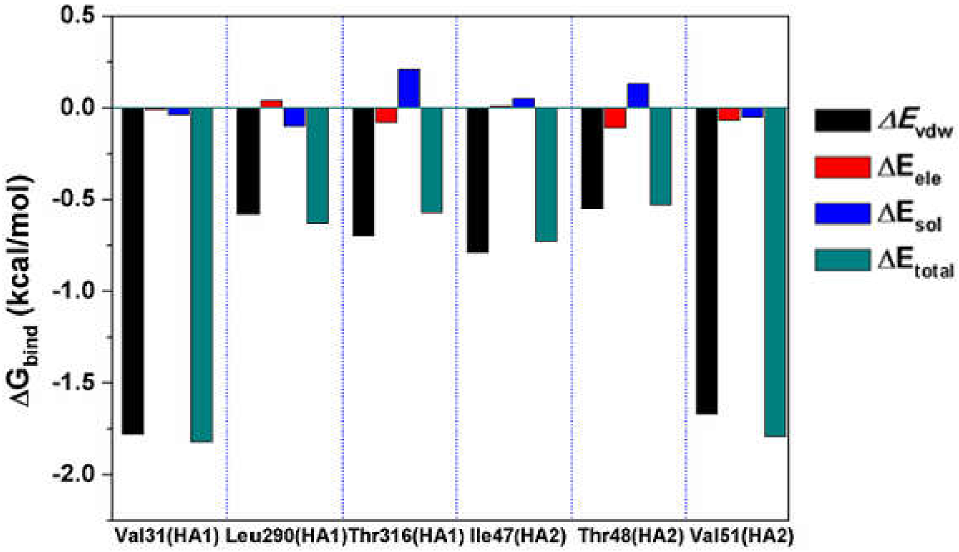

Figure S6 The comparation between the binding free energy with the first 100 ns and furthrer one with the extended 100 ns under neutral condition. (a) Snaps used in calculations extracted from the equilibrium stages within the first 100 ns. (b) Snaps used in calculations extracted from equilibrium stages within the second 100 ns.

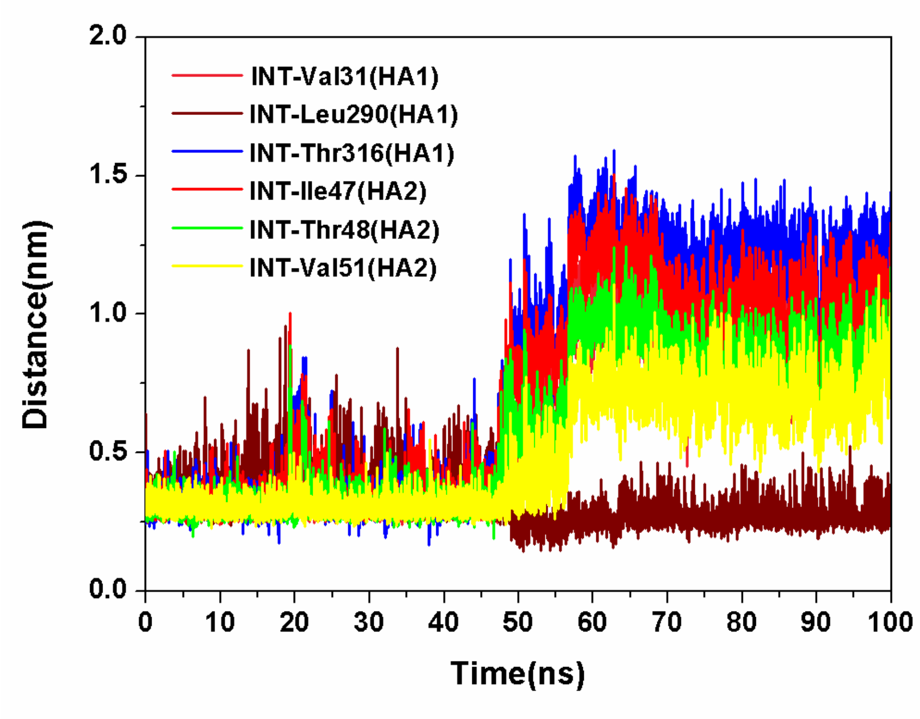

Figure S7 Distances between INT and the residues (HA1-Val31, HA1-Leu290, HA1-Thr316, HA2-Ile47, HA2-Thr48 and HA2-Val51) under acidic conditions from the repeated simulation.

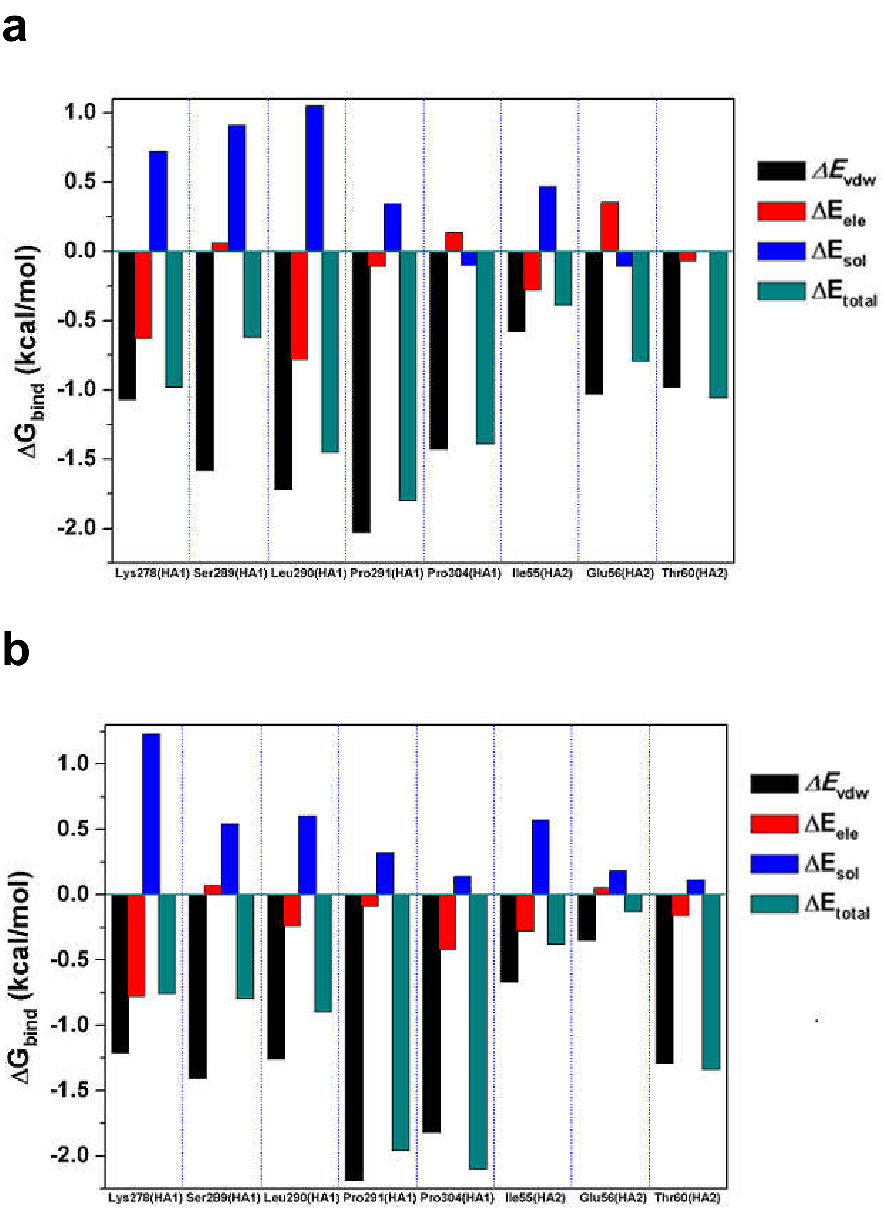

Figure S8 The comparation between the binding free energy with the first 100 ns and furthrer one with the extended 100 ns under acidic condition. (a) Snaps used in calculations extracted from the equilibrium stages within the first 100 ns. (b) Snaps used in calculations extracted from equilibrium stages within the second 100 ns.

Table S1. Protonation states of the titrable aminoacids of 1RUZ.

Subunit	Protonated residues
HA1	*all Arg, all Lys, Asp5, His12, His32, His41, Glu85, Asp101, Glu119, His183, His184, Glu216, His276, His286
HA2	*all Arg, all Lys, Gly1, His25, His26, Glu69, Glu74, Asp90, Glu97, Glu103, Glu105, Asp109, His111, Glu139, Glu150, Asp158

*Residues numbered according to the crystal structure of 1RUZ for easy understanding.

Table S2. The pKa of residues those close to binding site of INT-HA_{1ruz}.

Residues	pKa (with INT)	pKa (without INT)	Protonation state
*HA1-Glu302	4.43	4.43	deprotonated
*HA1-Lys278	10.60	10.60	protonated
*HA2-Glu56	3.54	3.49	deprotonated

*Residues numbered according to the main text.

Residues with the calculated pKa values higher than (or equal to) the investigated environment pH (pH = 4.5) were treated as protonated. The protonation state of the above residues is consistent with study by Zhou *et al*.
